# A simple monochromatic flow cytometric assay for assessment of intraerythrocytic development of *Plasmodium falciparum*

**DOI:** 10.1186/s12936-020-03156-1

**Published:** 2020-02-18

**Authors:** Kasem Kulkeaw, Nutpakal Ketprasit, Anchalee Tungtrongchitr, Duangdao Palasuwan

**Affiliations:** 1 Department of Parasitology, Faculty of Medicine Siriraj Hospital, Mahidol University, 2, Wanglang Road, Bangkoknoi, Bangkok 10700 Thailand; 2 Oxidation in Red Cell Disorders and Health Research Unit, Department of Clinical Microscopy, Faculty of Allied Health Sciences, Chulalongkorn University, 154 Rama 1 Road, Pathumwan, Bangkok 10330 Thailand

**Keywords:** Malaria parasite development, Monochromatic flow cytometric assay, *Plasmodium falciparum*, VSG

## Abstract

**Background:**

Gold standard microscopic examination of *Plasmodium falciparum* intraerythrocytic stage remains an important process for staging and enumerating parasitized erythrocytes in culture; however, microscopy is laborious and its accuracy is dependent upon the skill of the examiner.

**Methods:**

In this study, ViSafe Green (VSG), which is a nucleic acid-binding fluorescent dye, was used for assessing in vitro development of *P. falciparum* using flow cytometry.

**Results:**

Fluorescence intensity of VSG was found to depend on the developmental stage of parasites. Specifically, multiple-nuclei-containing schizonts were observed in the VSG^high^ population, and growing trophozoites and ring-shaped forms were observed in the VSG^intermediate^ and VSG^low^ populations. The efficacy of VSG-based assay was found to be comparable to the microscopic examination method, and it demonstrated an ability to detect as low as 0.001% of the parasitaemia estimated by Giemsa staining. Moreover, when applying VSG for anti-malarial drug test, it was able to observe the growth inhibitory effect of dihydroartemisinin, the front-line drug for malaria therapy.

**Conclusions:**

Taken together, the results of this study suggest the VSG-based flow cytometric assay to be a simple and reliable assay for assessing *P. falciparum* malaria development in vitro.

## Background

*Plasmodium falciparum* remains a wide-spreading and highly virulent parasitic protozoan worldwide [1]. The mortality rate is highest in tropical and subtropical areas. Despite the development of effective anti-malarial drugs, drug-resistant strains of malaria are reported annually [1, 2], which emphasizes the need for ongoing drug resistance surveillance, continued study of the underlying mechanisms of drug resistance, and novel drug development. Culture of laboratory strains or field-isolates of *P. falciparum* has been widely used for these investigations. Microscopic examination is an effective method for assessing *in vitro* growth of malaria parasites in *P. falciparum* culture, as well as for drug sensitivity testing [3–5]. Nevertheless, the counting of malaria-infected erythrocytes under a microscope is tedious and time-consuming. This method requires a well-trained and experienced microscopist to enumerate and differentiate various stages of malaria parasites. Inter-rater variability among microscopists is, therefore, a drawback of the microscopic examination method.

Flow cytometry facilitates quantitative analysis of cells at high-speed, at high sensitivity, and in a semi-automatic manner. Given that erythrocytes lack nuclear DNA, detection of malarial DNA in erythrocytes is one of the most common assays. Several DNA-binding fluorescent dyes (fluorochromes) are available. Some fluorochromes, including hydroethidine [6], ethidium bromide [7], propidium iodide [8], SYBR Green I [9, 10], YOYO-1 [11], Hoechst 33258 [12], and Hoechst 33342 [13], are employed to stain the DNA of the malaria parasite in erythrocytes. To use hydroethidine and Hoechst 33342, cells need to be incubated at 37 °C, which lengthens the processing time. The use of ethidium bromide has decreased due to its carcinogenic property. Propidium iodide was useful for evaluating invasion of merozoites into erythrocytes in anti-malarial drug test [8]. SYBR Green I facilitated quantification of parasitized erythrocytes at different stages of development [9]. However, propidium iodide, SYBR Green I, YOYO-1, and Hoechst require an extra step of cell membrane permeabilization for which aldehyde-based or ethanol-based fixation is often used. Since these methods alter cell structure, morphologic study of malaria parasite cannot be performed after their use.

Many nucleic acid-binding fluorochromes are commercially available and have been applied for visualizing DNA or RNA in agarose or polyacrylamide gel. ViSafe Green (VSG) is a stable, sensitive, and environmentally safe nucleic acid-binding fluorescent dye. VSG can be activated by a 250–300 nm wavelength (UV) and it emits spectra similar to that of ethidium bromide [14]. Thus, VSG is an alternative to ethidium bromide for visualizing DNA or RNA in agarose gel. Given the availability of new nucleic acid-binding fluorochromes, this study set forth to develop a simple and fixation-free method that uses VSG to enumerate malaria-infected erythrocytes, and to assess intraerythrocytic development in culture. In addition, its utility for anti-malarial drug susceptibility assay was demonstrated.

## Methods

### Parasite and culture

*Plasmodium falciparum* strain K1 was used in this study. Parasites were maintained as described previously [15]. Briefly, malaria culture medium (MCM) was prepared that consisted of RPMI 1640 (Sigma-Aldrich Corporation, St. Louis, MO, USA) supplemented with 5.96 g/L HEPES, 2 g/L sodium bicarbonate, and 10% heat-inactivated human AB serum. The parasites were cultured in a T-25 flask containing 5% human O+ erythrocytes in MCM in a 5% CO_2_ environment at 37 °C. To assess the developmental stages of the studied parasites, a thin blood smear was prepared on a glass slide. Cells were visualized by staining with Giemsa dye prior to observation under light microscope [16].

### Synchronization of *Plasmodium falciparum*-infected erythrocytes

Parasites were maintained in a synchronicity manner as described previously [17]. Briefly, parasites were allowed to grow to the ring stage, and they could not be older than 10 to 12 h after merozoite invasion. The parasite culture was spun down at 2000 revolutions per minute (rpm) for 5 min. After removal of the supernatant, an equal volume of sterile 5% d-sorbitol in distilled water was mixed with the packed erythrocytes and the mixture was incubated at 37 °C for 10 min. After incubation, the cell suspension was spun down at 2000 rpm for 5 min and then washed three times with RPMI 1640. The parasitaemia and synchronicity were evaluated by counting the infected cells per 1000 erythrocytes on a Giemsa-stained thin blood smear under a microscope. Ninety percent synchronicity was accepted for this experiment. The synchronized parasites were adjusted to 1% parasitaemia with fresh human O+ erythrocytes and cultured in MCM as described above.

### Preparation of *P. falciparum* gametocyte

Gametocytes were prepared as described previously [18]. Briefly, the parasites were allowed to grow to the ring stage at 3–5% parasitaemia in MCM and then adjusted to 1% ring-stage parasitaemia with fresh human O+ erythrocytes. To induce gametocyte formation, MCM was replaced with a gametocyte-inducing medium, which is MCM consisting of 0.37 mM hypoxanthine (Sigma-Aldrich) and 10% human AB serum without heat inactivation. A 75% volume of the gametocyte-inducing medium was replaced daily. To assess sexual development of *P. falciparum*, a thin blood smear was prepared on a glass slide and stained with Giemsa dye prior to observation under a light microscope. Gametocytes were identified as described in a published method [19].

### ViSafe Green staining and flow cytometric analysis

Given that no previous study has used VSG (20 mg/mL; Vivantis Technologies, Salangor, Malaysia) for nucleic staining in viable cells, the fluorescent dye concentration was initially optimized. Briefly, cells were suspended in diluted concentrations of VSG (0.5, 1, 2, 5, 10, and 20 μg/mL) in phosphate-buffered saline (PBS) and kept in the dark at room temperature (RT) for 20 min. Cells were then subjected to flow cytometric analysis and cell sorting using a FACS Aria II Instrument (BD Biosciences, San Jose, CA, USA) without cell washing. A threshold of FSC was set at 10,000 to reduce a contamination of cell debris (Additional file 1: Fig. S1). A type of VSG-activating laser and a suitable fluorescence detector of an emitted fluorescent signal were determined. Given that the FACS Aria II is equipped with 488-, 633-, and 375-nm lasers, all three lasers were used for VSG activation. Fluorescence detectors of FITC (500–560 nm), PE (543–627 nm), PE-Texas Red (593–639 nm), PerCP-Cy5-5 (655–735 nm), PE-Cy7 (720–840 nm), APC (640–680 nm), A700 (685–775 nm), APC-Cy7 (720–840), BV421 (400–500 nm), BV510 (500–560 nm), and BV605 (590–630 nm) were used for detection of the emitted fluorescent signals. The flow cytometric data were analysed using FlowJo version 10 software (Tree Star, Inc., Ashland, OR, USA). To increase the accuracy of flow cytometric analysis, non-single cells were excluded by gating according to forward scatter (FSC) and side scatter (SSC) characteristics of cells. Briefly, cells were first gated using forward scatter area (FSC-A) parameter at the X-axis, and then using forward scatter height (FSC-H) parameter at the Y-axis. Cells having the characteristic of FSC-A equal to FSC-H were gated. Then, side scatter width (SSC-W) and side scatter height (SSC-H) were set at the X-axis and Y-axis, respectively, in order to exclude cells having SSC-W^high^, which are not single cells. Cells were then further gated according to forward scatter width (FSC-W) and forward scatter height (FSC-H). Cells were sorted into PBS containing 1% fetal bovine serum (FBS) for morphologic analysis.

### Giemsa staining and microscopy

Cells were affixed to glass slides using a CytoSpinTM4 Cytocentrifuge (Thermo Fisher Scientific, Inc., Waltham, MA, USA) at 450 rpm for 7 min, and then rapidly air-dried. Cells were fixed with absolute methanol and stained using a 1:18 diluted Giemsa solution at RT for 30 min. After one wash with running tap water, slides were air-dried and covered with glass coverslips with one drop of mounting solution. Cell morphology was assessed using an Olympus BX53 using an objective lens at 100× magnification. For Giemsa-stained thin film of the culture, a minimum of 100 fields was examined at 100× magnification with oil immersion [20].

### Fluorescence microscopic imaging

To ensure that VSG is able to pass through the cell membrane and bind to the parasite’s nucleic acid, 50–100 μL of VSG-stained cells was dropped onto a glass slide and covered with a thin glass. The VSG-stained cells were observed under a laser-scanning confocal microscope (ECLIPSE Ti-Clsi4 Laser Unit; Nikon Corporation, Tokyo, Japan). Differential interference contrast and a 488-nm argon-ion laser was used for microscopic imaging.

### Reliability and sensitivity

To test the reliability of the VSG-based flow cytometric assay, the parasitaemia estimated from microscopic examination of the Giemsa-stained blood smear (the standard method) and the percentage of VSG+ cells obtained from flow cytometry were compared. Various concentrations of parasitaemia were prepared by diluting the parasitized erythrocytes in a 5% uninfected erythrocyte suspension. Spearman’s rank correlation coefficient was used to assess the strength of association between the standard microscopic assay and VSG-based flow cytometry. For sensitivity testing, culture of *P. falciparum* was diluted to 0.001% parasitaemia, which is the limit of detection in routine microscopic diagnosis [20], and then stained with VSG as describe above and analysed by flow cytometry.

### Lethal induction of *P. falciparum* using dihydroartemisinin

Dihydroartemisinin (DHA) (Sigma-Aldrich), which is a primary drug for falciparum malaria treatment, was used in this study to induce lethal form of the parasites.

DHA was prepared at a concentration of 700 nM in dimethyl sulfoxide (DMSO) (Sigma-Aldrich) as described in previous study [21]. In short, 2 mg of DHA was resuspended in 2 mL of DMSO and used as stock solution. The stock solution was then diluted fivefold in DMSO to achieve a drug concentration of 200 µg/mL (700 μM). Synchronized ring stages of *P. falciparum* K1 strain were diluted with 5% haematocrit O cell and MCM to obtain 1% parasitaemia and 2% haematocrit. Twenty µL of DHA solution (700 μM) or DMSO was mixed with 2 mL of MCM to obtain a concentration of 7 μM. Then, 100 µL of 7-μM DHA was mixed with 900 µL of the infected erythrocytes. Therefore, the final concentration of DHA in the culture was 700 nM. To examine dose-dependent effect of DHA, three different concentrations of DHA (350, 700 and 1400 nM) were prepared. The parasites were then exposed to DHA or DMSO (as control) in a 5% CO_2_ atmosphere at 37 °C for 24 h. To access time-dependent effect of DHA, the parasites were exposed to 700-nM DHA for 6 h and then cultured in MCM without DHA [21]. The parasites were collected at different time points (12, 24, 36, 48 and 60 h) and subjected to Giemsa dye stain and VSG-based flow cytometry as described above.

### Statistical analysis

Data analysis and graph generation were performed using GraphPad Prism software version 5.0 (GraphPad Software, Inc., San Diego, CA, USA). Results are expressed as mean ± standard deviation (SD) and coefficient of variation (CV). Spearman’s rank correlation coefficient was used to measure the strength of association between standard microscopy and VSG-based flow cytometry. Statistically significant differences were identified using non-parametric Student’s *t*-test. A *p*-value less than 0.05 was regarded as being statistically significant.

## Results

### Cell permeability of VSG dye

**Fig. 1:**
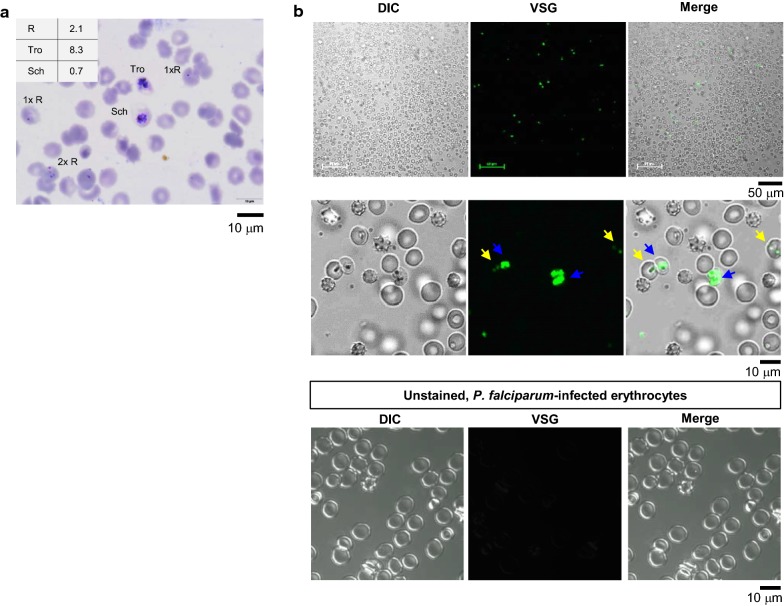
Cell permeability of VSG dye. **a** Giemsa-stained thin blood smear of the non-synchronized culture revealed mixed developmental stages of falciparum malaria parasites. Scale bars: 10 µm. **b** Laser scanning confocal microscopic images of *P. falciparum*-infected erythrocytes uptaking VSG (upper and middle panels). Differential interference contrast images are shown (far left panel). VSG was activated by a 488-nm argon-ion laser, and is displayed as green in the middle panel. Differential interference contrast and fluorescent images are merged in the far-right panel. Yellow and blue arrows indicate low and high intensity of VSG, respectively. The unstained, *P. falciparum*-infected erythrocytes were used as a control. Scale bars: 50 µm at upper and 10 µm at middle and lower panels. R, ring form; Tro, trophozoite; Sch, schizont; DIC, differential interference contrast; VSG, ViSafe Green

To ensure that VSG is cell-permeable and that it binds to nucleic acid, a non-synchronized culture of *P. falciparum* (Fig. 1a) was incubated with VSG dye without fixation and subjected to laser-scanning confocal microscope imaging in which the emitted fluorescent signal of VSG was displayed as green. To deny the possibility of autofluorescence, a sample of unstained *P. falciparum*-infected erythrocytes was used as a control. There was no green colour observed in the control (Fig. 1b, lower panels). At lower magnification, cells having green colour were observed, and they accounted for 1.9% of total observed cells (Fig. 1b, upper panels). Higher magnification images revealed green colour inside the erythrocytes (Fig. 1b, yellow and blue arrows in middle panels), which suggests cell membrane permeability of VSG. Moreover, the intensity of green colour was shown to vary, with intensity roughly grouped into low or high intensity (Fig. 1b, yellow and blue arrows, respectively). Two green dots were also observed in a single erythrocyte similar to those found in the Giemsa-stained thin blood smear, which suggests multiple infection of *P. falciparum*. These findings indicate that VSG was able to permeate the *P. falciparum*-infected erythrocytes.

### Optimization of VSG stain for flow cytometry

**Fig. 2:**
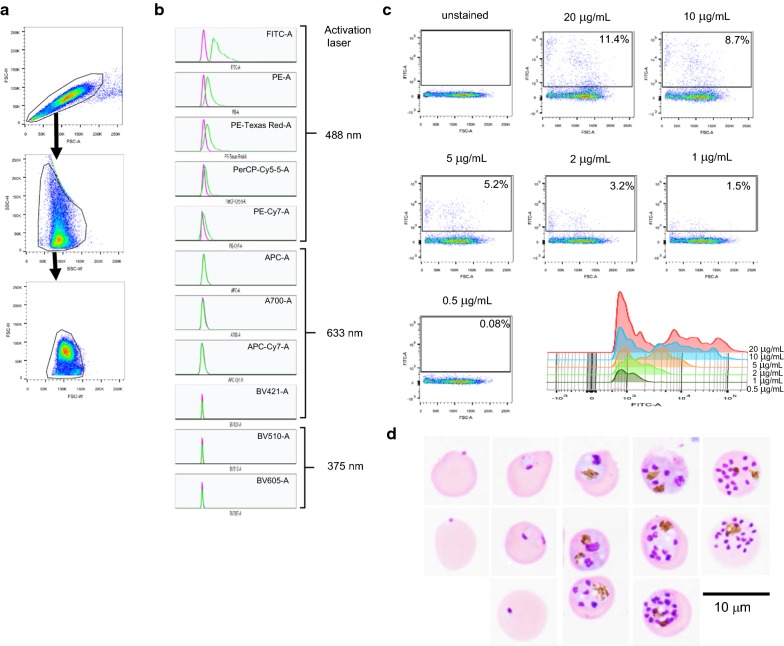
Optimization of VSG staining of *P. falciparum*-infected erythrocytes. **a** Gate setting for flow cytometric analysis. Non-single cells were excluded by gating according to FSC-H, FSC-W, SSC-H, and SSC-W. **b** Histograms show fluorescence intensity of VSG+ cells (green) excited by 488-nm, 633-nm, and 375-nm lasers. To read the emitted fluorescent signal, detectors of FITC (500–560 nm), PE (543–627 nm), PE-Texas Red (593–639 nm), PerCP-Cy5-5 (655–735 nm), and PE-Cy7 (720–840 nm) were used for 488-nm activating laser; detectors of APC (640–680 nm), A700 (685–775 nm), and APC-Cy7 (720–840) were used for 561-nm activating laser; and, detectors of BV421 (400–500 nm), BV510 (500–560 nm), and BV605 (590–630 nm) were used for 445-nm activating laser. Histogram of sample not stained with VSG was set as VSG negative (shown in magenta). **c** Representative flow cytometric profiles of samples stained with VSG at 0.5, 1, 2, 5, 10, and 20 µg/mL relative to the 10,000x concentration (20 mg/mL) of the commercial version. Overlaid histogram of VSG+ cells obtained from staining with different concentrations of VSG is shown on the left side of flow cytometric images. **d** Representative images of Giemsa-stained erythrocytes in VSG+ fraction acquired using an objective lens at 100×. Cells were sorted from the sample stained with 10 µg/mL of VSG. Scale bars: 10 µm. FSC-A, forward scatter area; FSC-H, forward scatter height; FSC-W, forward scatter width; SSC-W, side scatter width; SSC-H, side scatter height; DIC, differential interference contrast; VSG, ViSafe Green

Given that VSG has never been used for flow cytometry, a type of VSG-activating laser and a suitable fluorescence detector had first to be identified. The concentration of VSG was then optimized. In flow cytometry analysis, non-single cells were excluded by gating according to forward scatter (FSC) and side scatter (SSC) characteristics of cells. Briefly, cells were first gated using FSC-A parameter at the X-axis, and using FSC-H parameter at the Y-axis (Fig. 2a, upper panel). Cells having the characteristic of FSC-A equal to FSC-H were gated. Then, SSC-W and SSC-H were set at the X-axis and Y-axis, respectively (Fig. 2a, middle panel), in order to exclude cells having SSC-W^high^, which are not single cells. Cells were then further gated according to FSC-W and FSC-H (Fig. 2a, lower panel). Based on FSC-A and SSC-A, there were two populations: cells having FSC-A lower or higher than 50 K (Additional file 1: Fig. S1A). Both contained *P. falciparum*-infected and non-infected erythrocytes (Additional file 1: Fig. S1B). Thus, both populations were included for analysis. These initial gating steps aimed to obtain single cells, which increases the accuracy of flow cytometric analysis. Using a FACS Aria II, the 488-nm laser could activate VSG and effectuate emission of a fluorescent signal, whereas the 633-nm and 375-nm laser could not (Fig. 2b). When a detector of FITC fluorochrome (500–560 nm) was used, VSG+ cells (green-coloured lines) could be separated from the unstained cells (magenta-coloured lines). In contrast, when a detector of PE (543–627 nm) and PE-Texas Red (593–639 nm) was used, VSG+ cells (green-coloured lines) overlapped with the unstained cells (magenta-coloured lines), which limited our ability to analyse the parasitized cells. Therefore, it was decided to use the 488-nm laser for VSG activation, and the FITC detector to read the emitted fluorescent signal.

To determine the optimal concentration of VSG, *P. falciparum*-infected erythrocytes were incubated with 0.5, 1, 2, 5, 10, and 20 μg/mL of VSG. The optimal VSG concentration was determined based on its ability to fractionate *P. falciparum*-infected erythrocytes from non-infected cells. As shown in Fig. 2c, 20 and 10 μg/mL of VSG were the concentrations that yielded the highest fluorescence intensity in VSG+ cells. Moreover, different intensity of fluorescence was observed in the 20 and 10 μg/mL VSG-stained samples (Fig. 2c, histogram), which is a finding that is consistent with confocal microscopic data. The 200, 100, and 50 μg/mL VSG concentrations were excluded due to an upward shift in the dots on the flow cytometric profile, which suggested an increase in non-specific staining (high background). Microscopic observation of sorted VSG+ cells showed that 10 μg/mL of VSG yielded all stages of intraerythrocytic development of *P. falciparum* (Fig. 2d). In agreement with Fig. 2d, Giemsa staining of presorted sample showed 10.4% parasitaemia that consisted of 9.8% ring form, 0.1% trophozoites, and 0.5% schizonts, which strongly suggests the accuracy of VSG at a concentration of 10 μg/mL. Therefore, 10 μg/mL of VSG was used for other experiments in this study.

### Validation of the VSG staining method

**Fig. 3:**
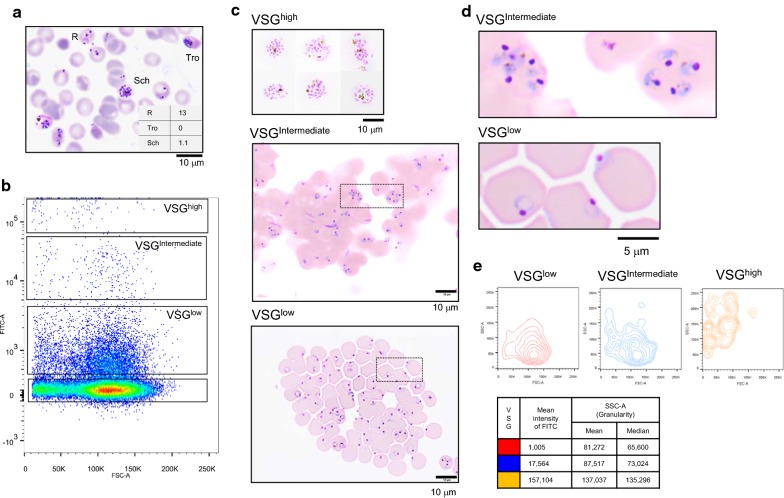
Intensity of VSG depends on the stage of intraerythrocytic development of *P. falciparum.***a** Giemsa-stained thin blood smear of the non-synchronized culture revealed mixed developmental stages of falciparum malaria parasites. **b** Flow cytometric profile of *P. falciparum* infected erythrocytes. The non-synchronized culture of malaria parasites was stained with 10 µg/mL of VSG. Single cells were separated based on fluorescence intensity into high, intermediate, or low (hereafter referred to as VSG^high^, VSG^intermediate^, and VSG^low^, respectively). **c** Morphology of Giemsa-stained VSG^high^, VSG^intermediate^, and VSG^low^ cells. **d** Higher magnification of *P. falciparum*-infected cells in the boxed area of VSG^intermediate^ and VSG^low^ cells in Fig. 3c. **e** Cell granularity of VSG^high^, VSG^intermediate^, and VSG^low^ cells was assessed based on SSC-A. Scale bars: 10 µm for Fig. 3a, c, and 5 µm for Fig. 3d. *FSC-A*, forward scatter area; VSG, ViSafe Green

To test that each stage of intraerythrocytic development of *P. falciparum* could be fractionated based on the intensity of VSG, a non-synchronized culture of malaria parasites was prepared. As a standard method, Giemsa staining of thin blood film showed 14% parasitaemia that consisted of 13% ring form, 0% trophozoites, and 1.1% schizonts (Fig. 3a). The VSG+ cells were separated according to intensity into low, intermediate, or high (hereafter referred to as VSG^low^, VSG^intermediate^, and VSG^high^, respectively) (Fig. 3b), and their morphologies were examined. Schizonts were observed only in VSG^high^ fraction, and ring forms and growing trophozoites were observed only in VSG^intermediate^ and VSG^low^ fraction (Fig. 3c). Moreover, different morphology of the *P. falciparum* parasites could be observed in VSG^intermediate^ and VSG^low^ fraction. The cytoplasm of *P. falciparum* in the VSG^intermediate^ fraction was thicker than that in the VSG^low^ fraction, and it contained malarial pigment (Fig. 3d). These findings were in agreement with microscopically examined Giemsa-stained thin blood film that revealed ring form, trophozoites, and schizonts in the culture, which suggested that this protocol was optimal. Thus, fluorescence intensity of VSG depends on the stage of *in vitro* malaria development.

To test whether VSG-based flow cytometric analysis could distinguish gametocytes from schizonts, *P. falciparum* strain K1 was grown in gametocyte-inducing culture medium and performed VSG-based flow cytometric analysis. Cells in VSG^low^, VSG^intermediate^, and VSG^high^ fraction were sorted and stained with Giemsa. In the VSG^high^ fraction, parasitized erythrocytes could be observed having granular distribution of haemozoin that resembled stage-IB gametocytes. Moreover, some were elongated and D-shaped within erythrocytes, which are key characteristics of stage-II gametocytes. Early schizonts having 2 and 6 divided nucleus, and mature schizonts consisting of 14 merozoites were also observed in the VSG^high^ fraction, whereas ring forms and trophozoites were observed in the VSG^low^ and VSG^intermediate^ fractions, respectively (Additional file 2: Fig. S2). Thus, VSG-based flow cytometric assay is not able to distinguish gametocytes from schizonts.

Given the ability of VSG to differentiate intraerythrocytic stages, the study explored whether change in cell granularity is related to the developmental stages of *P. falciparum*. VSG^low^, VSG^intermediate^, and VSG^high^ cells were gated and analysed for SSC-A, which is an indicator of cell granularity. As shown in Fig. 3e, the median of SSC-A increased about 2 times when VSG^low^ and VSG^intermediate^ cells developed into VSG^high^ cells. These results suggest that change in cell granularity is related to intraerythrocytic development of *P. falciparum,* and that this change can be assessed using VSG-based flow cytometry.

### Linearity and sensitivity of the VSG-based flow cytometric assay

**Fig. 4:**
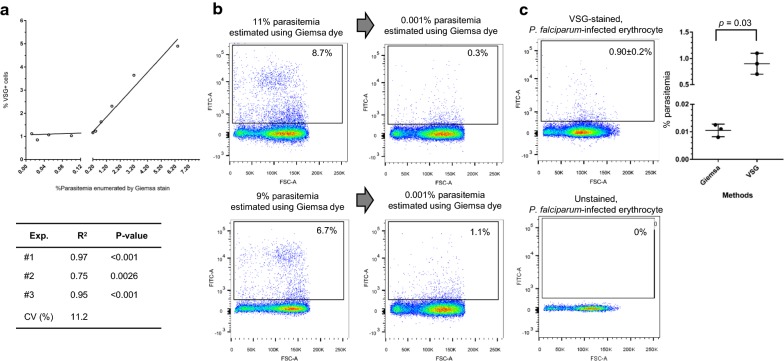
Linearity and sensitivity of the VSG-based flow cytometric assay. **a** Representative graph of the Spearman’s rank correlation coefficients. Percentage of *P. falciparum*-infected erythrocytes was obtained from VSG-based flow cytometry (Y-axis) and from Giemsa staining of thin blood film (X-axis). Three independent analyses were performed that revealed a range of infected erythrocytes of 0.01–6.4%, as shown in the table. **b** Sensitivity of VSG-based flow cytometry. Culture of *P. falciparum* was diluted to 0.001% parasitaemia, which is the limit of detection in routine microscopic diagnosis [20], and then analysed by flow cytometry. Representative flow cytometric profile and data are shown as mean ± SD. The graph shows a comparison of parasitaemia detected by standard microscope and VSG+ cells detected by flow cytometry. **c** Reproducibility of the VSG-based flow cytometric assay for low parasitaemia culture. Three independent settings of *P. falciparum* culture were diluted to 0.01% parasitaemia and analysed using flow cytometry. Unstained, *P. falciparum*-infected erythrocytes were used as control. VSG, ViSafe Green; FSC-A, forward scatter-area

To evaluate the optimized protocol relative to its ability to enumerate parasitized erythrocytes, detection of malaria-infected erythrocytes was performed in a dose-dependent manner. Various concentrations of malaria-infected erythrocytes were prepared. Two-fold dilutions of infected cells were prepared using non-infected erythrocytes as diluent. That analysis revealed that VSG-based flow cytometry could detect malaria-infected erythrocytes in a dose-dependent manner (Fig. 4a). The relative values correlated well between the two assays (*r*^2^ = 0.75–0.97; *p* < 0.05). The same results were observed from three independent experiments (CV = 11.2%), indicating the reproducibility of linearity measurement.

To assess the sensitivity of VSG-based flow cytometry, parasitized erythrocytes were diluted to 0.001%, which is the limit of detection in routine microscopic diagnosis [20]. As shown in Fig. 4b, two independent cultures were analysed for each cytometry run. There were 11% and 9% parasitaemia enumerated using Giemsa-based microscopy. The parasites were diluted to 0.001% using non-infected erythrocytes as diluent. The diluted samples having 0.001% parasitaemia were then subjected to flow cytometry analysis. VSG-based flow cytometry was capable of detecting 0.3% and 1.1% of VSG+ cells, which is 300–1000 times higher than the detection rate (0.001% parasitaemia) by Giemsa-based microscopy. Next, the reproducibility of the developed assay for enumeration of low parasitaemia was examined. Three independent settings of malaria culture was prepared and diluted them to 0.01% parasitaemia, which is a minimum value that correlated well with standard microscopic examination (Fig. 4a). All three independent runs of VSG-based flow cytometry were able to detect 0.9 ± 0.2% of VSG+ cells (CV = 22%, Fig. 4c), implying reproducibility comparable to Giemsa-based microscopy (CV = 21.8%) for detection of low parasitaemia.

To examine the variability of VSG-based flow cytometric assay for enumeration and identification of *P. falciparum*-infected erythrocytes among different sets of parasite culture, parasite culture was prepared on different dates and compared the enumerated values of parasitized cells (mean ± SD) obtained from Giemsa-based microscopic analysis with those obtained from VSG-based flow cytometric analysis (Table 1). There were two types of culture: ring-form and trophozoite predominant. In both types of culture, the CV of the VSG-based flow cytometric assay for enumeration of parasitaemia was relatively lower than that of the microscopic method, which implies lower variability of the VSG-based flow cytometric assay. When analysing the variability of assays according to developmental stage, high CV values were obtained from both Giemsa-based microscopy and VSG-based flow cytometry, which is likely due to low parasitaemia in each developmental stage. Collectively, VSG-based flow cytometry is a reliable, sensitive, and reproducible assay for enumeration of parasitaemia.

**Table 1:** Comparison of a standard optical microscope and VSG-based flow cytometer for enumeration and identification of *P. falciparum*-infected erythrocytes

Ring-form predominant culture	Enumerated value of parasitized erythrocyte					
	Microscope			VSG-based flow cytometer		
	Mean	SD	CV (%)	Mean	SD	CV (%)
Ring-forms	11.5	2.3	20.4	10.4	3.7	35.4
Trophozoites	0.1	0.1	141.4	1.7	1.6	91.5
Schizonts	0.8	0.4	53.0	0.5	0.3	52.1
All stages	12.3	2.7	21.8	12.5	1.8	14.1

Trophozoite predominant culture	Enumerated value of parasitized erythrocyte					
	Microscope			VSG-based flow cytometer		
	Mean	SD	CV (%)	Mean	SD	CV (%)
Ring-forms	1.1	1.5	141.4	2.7	0.2	8.0
Trophozoites	8.5	0.3	3.3	4.4	0.4	9.6
Schizonts	0.4	0.5	141.4	0.1	0.04	60.6
All stages	9.9	1.7	17.1	7.1	0.7	9.5

### Application of VSG-based flow cytometry for synchronicity assessment and drug sensitivity testing

**Fig. 5:**
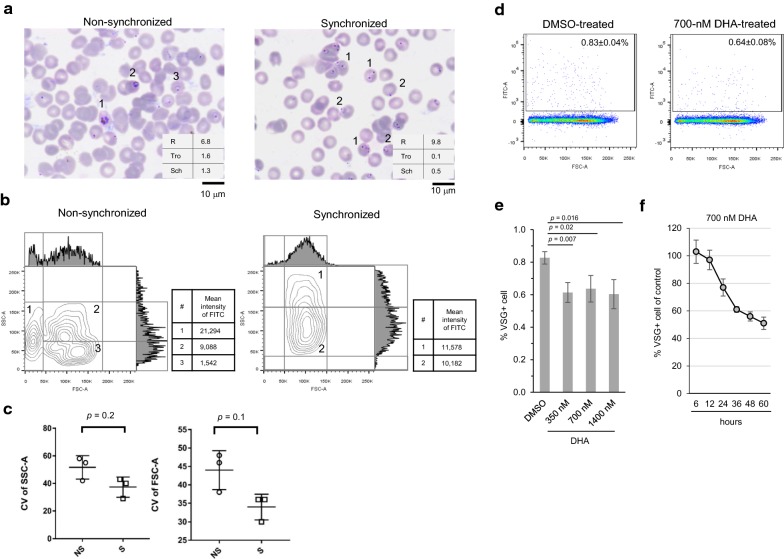
Application of VSG-based flow cytometry in synchrony assessment and anti-malarial drug assay. **a** Microscopic images of *P. falciparum*-infected erythrocytes derived from non-synchronized and synchronized cultures and stained with Giemsa dye. **b** Flow cytometric profiles of VSG+ cells are displayed according to FSC-A (X-axis) and SSC-A (Y-axis) in contour plots. Histograms of FSC-A and SSC-A are shown at the top and left side of the contour plots, respectively. There were at least three distinct populations observed in the non-synchronized culture (left panel), as follows: (1) cells having small size with various granularity; (2) cells having a relatively large size with high granularity; and, (3) cells having a relatively larger size with low granularity. There were two populations observed in the synchronized culture (right panel), as follows: (1) minor population with SSC-A higher than 160 K, and (2) major population with SSC-A lower than 160 K. **c** Graph of coefficient of variation of FSC-A and SSC-A. Dots represent three independent experiments. **d***P. falciparum*-infected erythrocytes were treated with anti-malarial drug dihydroartemisinin (DHA) and subjected to VSG-based flow cytometric analysis. Synchronized ring forms were prepared. **e** Dose-dependent effect of DHA treatment on growth inhibition of *P. falciparum*. The infected erythrocytes were treated with 350, 700, and 1400 nM of DHA for 24 h and subjected to VSG-based flow cytometric analysis. **f** Time-dependent effect of DHA treatment on growth inhibition of *P. falciparum*. After 6-h treatment with 700 nM DHA, the infected erythrocytes were cultured in MCM without DHA and collected at 12, 24, 36, 48, and 60 h for VSG-based flow cytometric analysis. The number of VSG+ cells in DMSO treatment was set as the control for each time point. The percentage of VSG+ cells in DHA treatment was compared to that observed in the control (Y-axis). X-axis indicates time of culture. Data represent mean ± SD of three independent experiments. FSC-A, forward scatter-area; SSC-A, side scatter-area; NS, non-synchronized culture; S, synchronized culture; CV, coefficient of variation

Synchronization of *P. falciparum* development is a common method used in routine culture, and its aim is to obtain a predominant intraerythrocytic stage of parasites. To explore whether VSG-based flow cytometry is capable of assessing synchronicity of *P. falciparum* development in a routine culture, synchronized and non-synchronized cultures of *P. falciparum* were prepared (Fig. 5a), stained with VSG, and subjected to flow cytometry analysis. Given the ability of flow cytometry to detect cell size and granularity using respective FSC and SSC, it was hypothesized that synchronized parasites have the same size and granularity, which suggests homogeneity. Thus, a quantile contour plot, which is an effective way to visualize distinct populations regardless of the numbers of cells displayed [22], was selected to assess cell homogeneity. In Fig. 5b, only VSG+ cells were displayed based on their size (as indicated by FSC-A on the X-axis) and granularity (as indicated by SSC-A on the Y-axis). To enhance the visualization of a distinct cell population having various cell size and granularity, histograms of FSC-A and SSC-A are also shown at the top and left side of the contour plots, respectively. Given the ability of contour plot to visualize cells based on the relative frequencies of sub-populations, distinct populations of VSG+ cells were able to be located using vertical and horizontal lines drawn on the contour plots. There were at least three distinct populations observed in the non-synchronized culture (Fig. 5b, left panel), as follows: (1) cells having small size with various granularity (approximately 0–45 K of FSC-A, and 30–170 K of SSC-A); (2) cells having a relatively large size with high granularity (approximately 45–185 K of FSC-A, and 75–170 K of SSC-A); and, (3) cells having a relatively larger size with low granularity (approximately 45–185 K of FSC-A, and 20–75 K of SSC-A). In contrast, only one minor (indicated as 1) and one major (indicated as 2) population were observed in the synchronized culture. They had a similar size (50–150 K of FSC-A), but different levels of granularity (35–240 K of SSC-A) (Fig. 5b, right panel). In the left panel of Fig. 5b, the population of VSG+ cells having less than 45 K of FSC-A was observed only in the non-synchronized culture (indicated as 1), but not in the synchronized culture (Fig. 5b, lower panel). Based on the intensity of VSG and microscopic images (Additional file 3: Fig. S3A), the population number 1, 2, and 3 in the left panel of Fig. 5b are schizonts, trophozoites, and ring forms, respectively. In contrast to observation in the non-synchronized culture, the minor and major populations of the synchronized culture could be separated based on SSC-A, as follows: (1) minor population with SSC-A higher than 160 K, and (2) major population with SSC-A lower than 160 K (Fig. 5b, right panel). Compared to the non-synchronized culture, a population of VSG+ cells having SSC-A higher than 160 K was observed only in the synchronized culture (indicated as 1 in Fig. 5b, lower panel). Based on microscopic images (Additional file 3: Fig. S3B), the VSG+ cells with more than 160 K SSC-A are infected erythrocytes containing multiple (60%) and single (40%) ring forms, and they had VSG intensity of 11,578; whereas, the VSG+ cells with lower than 160 K SSC-A are infected erythrocytes with multiple (35%) and single (65%) ring forms. Disappearance of the population having more than 160 K of SSC-A on the contour plot of the non-synchronized culture (Fig. 5b, left panel) and less than 45 K of FSC-A on the contour plot of the synchronized culture (Fig. 5b, right panel) resulted from different developmental stage of *Plasmodium* between the two separate cultures. To confirm heterogeneity in the non-synchronized culture, the CV, which is a measure of relative variability, of FSC-A and SSC-A was statistically analysed. Despite statistical non-significance (*p* > 0.05), the non-synchronized culture tended to have a higher CV for both FSC-A and SSC-A (Fig. 5c), which confirms the relatively high heterogeneity of VSG+ cells. Thus, VSG-based flow cytometry is an effective alternative method for assessing synchronicity of *P. falciparum* development in erythrocytes.

To demonstrate the use of VSG for assessment of growth inhibitory effect of anti-malarial drug, malaria-infected erythrocytes were incubated with 700 nM DHA for 24 h following a standard assay [21]. The DHA- and DMSO-treated cells were stained with VSG and analysed by flow cytometry. In both the presence and absence of the drug, there were VSG+ cells exhibiting VSG^intermediate^ and VSG^low^ (Fig. 5d), which were likely to resemble trophozoite and ring form, respectively. These results showed that the number of VSG+ cells decreased following DHA treatment (Fig. 5d, right panel) compared to that of the DMSO-treated control cells (Fig. 5d, left panel and *p* = 0.02). The majority of DHA-treated VSG+ cells appear as VSG^low^, implying that ring form was predominant. In contrast, both VSG^intermediate^ and VSG^low^ cells were observed in the DMSO-treated control (Fig. 5d, left panel), implying that both ring from and trophozoite were present in the culture. Similar to 700-nM DHA treatment, number of VSG+ cells also decreased after treatment with 350 and 1400 nM DHA for 24 h (*p* = 0.007 and 0.016, respectively); however, there was no difference in number of VSG+ cells among doses (Fig. 5e). Moreover, VSG-based flow cytometry was able to access effect of 700-nM DHA in time-dependent manner (Fig. 5f). According to the VSG-based flow cytometric data, DHA likely inhibited parasite growth. Therefore, the VSG-based flow cytometric assay can be used as an alternative assay for assessment of *P. falciparum* growth in the presence of anti-malarial drug *in vitro*.

## Discussion

Many flow cytometric assays have been developed to detect the malaria parasite; however, these assays are complicated, time consuming, and/or insufficiently sensitive. In this study, VSG was used to detect and purify *P. falciparum*-infected erythrocytes using flow cytometry. VSG could enter and bind to the nucleic acid of ring form, trophozoite, schizont and early stage of gametocytes of *P. falciparum* growing in culture. Twenty-minute incubation at room temperature without fixation makes this method faster and simpler to perform than other malaria detection assays.

The degree of parasitaemia detected by VSG-based flow cytometry was different from the degree of parasitaemia detected by the standard microscopic method. This difference is likely due to the method of cell analysis. It is recommended that 10,000 cells be counted under the microscope in order to accurately quantify the number of malaria parasite-infected cells. In contrast, hundreds of thousands of cells could be analysed by flow cytometry. Thus, different percentages of infected cells could be expected from these two different methods. This difference between methods may be explained by the sensitivity of VSG dye and its ability to correctly identify different stages of *P. falciparum*. Further validation by comparing VSG to other florescent dyes is warranted. Even though the percentages of infected cells was different between methods, the VSG-based flow cytometric assay was found to be reliable for detecting the malaria parasite in a dose-dependent manner, and it was able to detect parasitized cells as low as 0.001%, which is the detection threshold for the standard microscopic method [20].

In addition to the higher percentage of infected cells identified by the VSG method, the fixation-free flow cytometric method profiled in this report also provides morphologic information. This is the first study to report the high specificity of a method by showing the morphology of fluorochrome-binding cells. VSG+ cells were infected by *P. falciparum*, and all four major stages of malaria parasite could uptake VSG. Moreover, the intensity of VSG was found to be commensurate with the amount of DNA and RNA, and the VSG intensity of schizonts was higher than that of ring-shaped forms.

VSG is commercially available for nucleic acid detection in agarose gel electrophoresis and it is difficult for VSG to permeate cells; however, the reported data shows that VSG could enter *P. falciparum*-infected erythrocytes. It needs to be further investigated whether the permeability of VSG is due to increased membrane transport for nucleosides, amino acids, and carbohydrates, as described in previous studies [23, 24].

Compared to other DNA-binding fluorochromes, VSG is more suitable for analysing malaria parasites for the following reasons. First, VSG can rapidly enter cells and bind to nucleic acid at ambient temperature. As such, there is no need for cell permeabilization, which shortens the pre-flow cytometry process. Second, the fluorescence emission spectrum of VSG is similar to spectra of FITC, the most widely used fluorochrome. This factor facilitates application of VSG with other flow cytometry. Third and last, although Hoechst is frequently used as nuclear DNA stains, these fluorochrome probes cannot be used when the cytometer being used does not have a UV or 405-nm laser. Thus, VSG can be used as an alternative choice in the aforementioned setting.

VSG is an attractive alternative in flow cytometric assay due to its speed and ease of use compared to that of other DNA-binding fluorochrome probes, including hydroethidine [6], ethidium bromide [7], propidium iodide [8], SYBR Green I [9, 10], YOYO-1 [11], Hoechst 33258 [12], and Hoechst 33342 [13]. In comparison with Coriphosphine O [25], which is a fluorochrome activated by 488-nm laser, the use of VSG is simpler and there is no requirement for incubation at 37 °C and cell washing prior to flow cytometric analysis. Moreover, the cost of VSG is lower than that of Coriphosphine O. Even though VSG is comparable to that of SYBR Green I relative to cost and ease of use (without cell fixation), the resolution of the different *Plasmodium* stages stained with SYBR Green I was not sharp [26], which had the effect of limiting parasite stage identification. Moreover, an additional step of cell fixation using paraformaldehyde (PFA) or 1% glutaraldehyde was reported [9]. Importantly, VSG-based flow cytometric assay was able to detect a lower percentage of parasitaemia than Coriphosphine O and SYBR Green I. Despite these advantages, the protocol still requires further optimization, and a comparison with the aforementioned dyes is needed to draw definitive conclusions, regarding the advantages and disadvantages of each dye relative to VSG. Moreover, since VSG was originally used for DNA or RNA stain in agarose gel, and the dye can be activated using a 254-nm UV transilluminator, it may be possible that VSG-stained cells can be analysed using a UV laser-equipped flow cytometer and cell sorter.

The accurate detection and quantification of *P. falciparum*-infected erythrocytes using an automated Sysmex Haematology Analyzer XN-30 was recently reported [27, 28]. This automatic machine employs a 405-nm laser to detect cells that need to be partially lysed to increase the permeability of florescent dye before cell analysis. Thus, this method limits morphological observation of parasitized cells after cell analysis. Importantly, only a specific florescent dye can be used with this cell analyzer. In contrast, VSG is commercially available and is activated using a 488-nm excitation laser, which is one of the most common lasers built into cell analyzers, including: FACSCalibur, FACS Aria (BD Bioscineces), ZE5 Cell Analyzer (Bio-Rad), CellSimple™ Cell Analyzer (Cell Signaling Technology), and Guava^®^ easyCyte™ Systems (Luminex). However, VSG-based flow cytometry is not able to distinguish early gametocyte stage in cultures containing schizonts.

Anti-malarial drug susceptibility assay is very useful for identifying pharmacologically active compounds, to monitor drug resistance, and to investigate the mechanisms underlying drug resistance. Effect of anti-malarial drugs is mainly characterized by the inhibition of parasite growth or maturation and multiplication. These parameters are often measured by uptake of radioisotope [H^3^] hypoxanthine into nucleic acid [29], enzymatic assay of *P. falciparum*-specific lactate dehydrogenase [30], or detection of *P. falciparum*-specific antigen histidine-rich protein 2 in the culture [31]. The utility of VSG for assessing the pharmacologic effect of anti-malarial drugs on organism development was demonstrated.

## Conclusions

Given a relative ease of use of fluorescent dyes, VSG-based flow cytometry may be an effective alternative assay for enumeration of parasitaemia, assessment of intraerythrocytic development and synchronicity, and anti-malarial drug effect.

## Supplementary information

**Additional file 1: Fig. S1.** Effect of cell debris on analysis of VSG+ cells by flow cytometry. (A) In flow cytometry, cell debris has a lower level of forward scatter (FSC), and it can be observed at the bottom left corner of the density plot [32]. To remove cell debris, the threshold of FSC was set at 10,000 for all experiments. The presence of debris in three independent experiments was examined and shown. Cells having characteristics of FSC^low^ and that are located at bottom left (circles) of the density plot are considered debris. There is 0.64±0.2% of debris, suggesting that the majority of analysed cells are not debris. Moreover, the presence of cell debris in VSG+ cells was checked by examining VSG+ cells according to FSC-A and SSC-A (lower panels). There was 0.69±0.13% of VSG+ cells that had characteristics of FSC^low^ at bottom left corner of the density plot. Therefore, more than 95% of cells analysed by VSG-based flow cytometry were unlikely to be cell debris. (B) Morphology of cells having characteristics of FSC^low^ (< 50 K). *P. falciparum*-infected erythrocytes were subjected to flow cytometric analysis. The gated single cells were sorted according to intensity of FSC. There were ring forms and schizonts observed in the FSC^low^ cells. Thus, inclusion of the FSC^low^ cells is required. Microscopic images were captured using a microscope with objective lens of 100x magnification.

**Additional file 2: Fig. S2.** VSG-based flow cytometric analysis of gametocyte. Early schizonts and mature schizonts (left panel) and early stage gametocytes (right panel) were observed in VSG^high^ fraction. In the VSG^high^ fraction, parasitized erythrocytes have granular distribution of haemozoin, resembling stage IB. Moreover, some were elongated and D-shaped within erythrocytes, which are key characteristics of stage II gametocytes. Early schizonts having 2 and 6 divided nucleus, and mature schizonts consisting of 14 merozoites were also observed in the VSG^high^ fraction, whereas ring forms and trophozoites were observed in the VSG^low^ and VSG^intermediate^ fractions. Scale bar: 10 μm.

**Additional file 3: Fig. S3.** Giemsa stain of VSG+ cells sorted from non-synchronized and synchronized culture of *P. falciparum*. (A) Morphology of three distinct populations observed in the non-synchronized culture (Fig. 5b, left panel): (1) cells having small size with various granularity (approximately 0–45 K of FSC-A, and 30–170 K of SSC-A); (2) cells having a relatively large size with high granularity (approximately 45–185 K of FSC-A, and 75–170 K of SSC-A); and, (3) cells having a relatively larger size with low granularity (approximately 45–185 K of FSC-A, and 20–75 K of SSC-A). Based on the intensity of VSG and microscopic images, the population number 1, 2, and 3 in the upper panel of Fig. 5b are schizonts, trophozoites, and ring forms, respectively. (B) Morphology of one minor (indicated as 1) and one major (indicated as 2) population observed in the synchronized culture (Fig. 5b, right panel): (1) cells having high granularity (more than 160 K of SSC-A); and (2) cells having with low granularity (lower than 160 K of SSC-A). Both had a similar size (50–150 K of FSC-A). Based on microscopic images, majority of the VSG+ cells with more than 160-K SSC-A are infected erythrocytes containing multiple ring forms, and they had VSG intensity of 11,578; whereas, the majority of VSG+ cells with lower than 160 K SSC-A are infected erythrocytes with single ring forms and they had VSG intensity of 10,182.

## Data Availability

The datasets used and/or analysed during the current study are available from the corresponding authors on reasonable request.

## Abbreviations

CV: Coefficient of variation
DHA: Dihydroartemisinin
DIC: Differential interference contrast
DMSO: Dimethyl sulfoxide
FBS: Fetal bovine serum
FSC-A: Forward scatter area
FSC-H: Forward scatter height
FSC-W: Forward scatter width
MCM: Malaria culture medium
NS: Non-synchronized
RPM: Revolutions per minute
RPMI: Roswell Park Memorial Institute
S: Synchronized
SD: Standard deviation
SSC-H: Side scatter height
SSC-W: Side scatter width
VSG: ViSafe Green
