# Supplementary figures and images for: A simple monochromatic flow cytometric assay for assessment of intraerythrocytic development of Plasmodium falciparum
Source: Malar J. 2020 Feb 18;19:74. doi: 10.1186/s12936-020-03156-1 (PMC7027058; doi:10.1186/s12936-020-03156-1)

**A**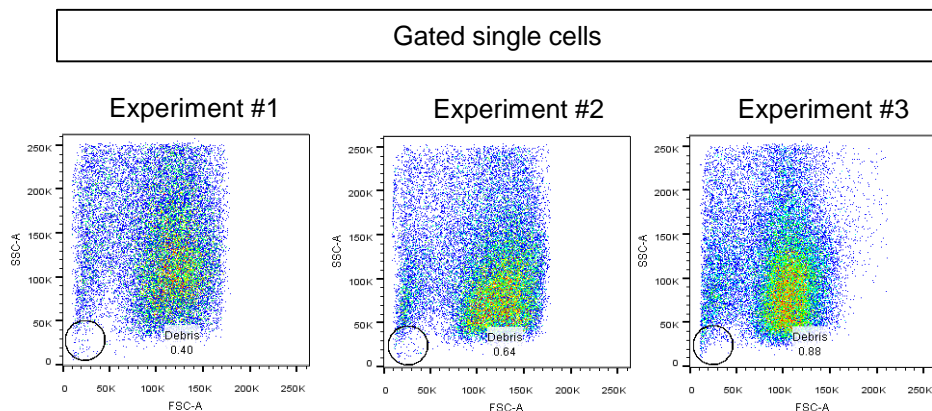

Mean of debris percentage =  $0.64 \pm 0.2\%$

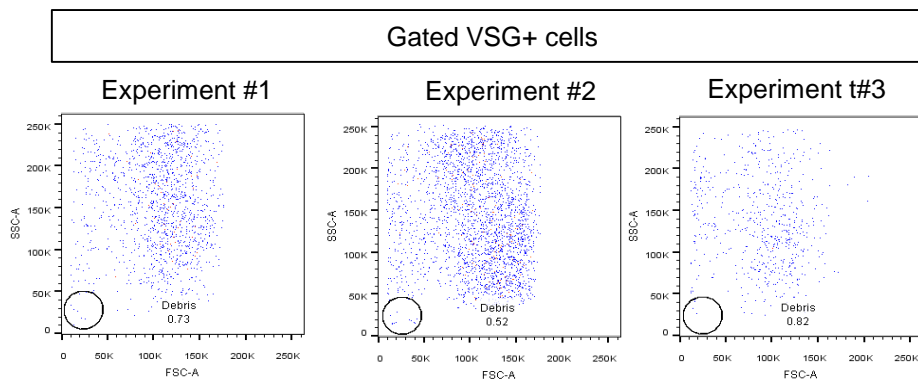

Mean of debris percentage =  $0.69 \pm 0.13\%$

**B**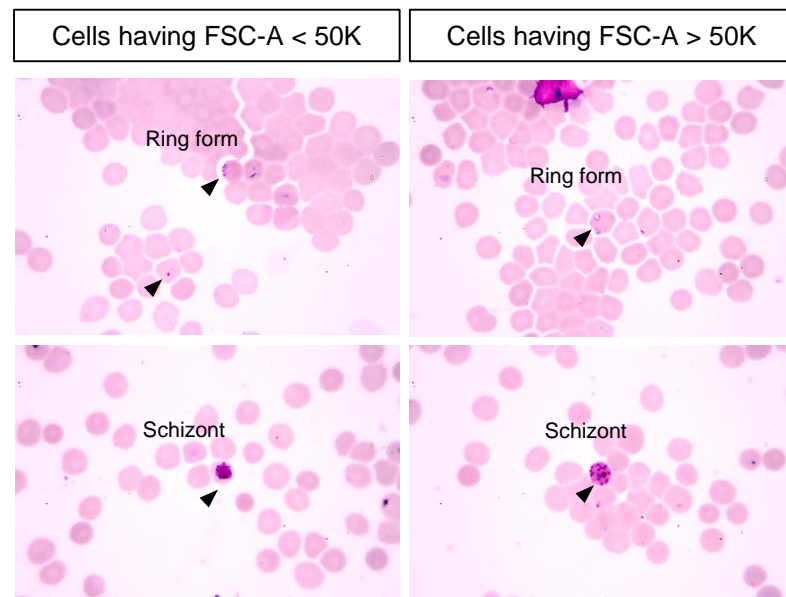

100X magnification

Supplement: Supplementary file 1 — Additional file 1: Fig. S1. Effect of cell debris on analysis of VSG+ cells by flow cytometry. (A) In flow cytometry, cell debris has a lower level of forward scatter (FSC), and it can be observed at the bottom left corner of the density plot [32]. To remove cell debris, the threshold of FSC was set at 10,000 for all experiments. The presence of debris in three independent experiments was examined and shown. Cells having characteristics of FSClow and that are located at bottom left (circles) of the density plot are considered debris. There is 0.64±0.2% of debris, suggesting that the majority of analysed cells are not debris. Moreover, the presence of cell debris in VSG+ cells was checked by examining VSG+ cells according to FSC-A and SSC-A (lower panels). There was 0.69±0.13% of VSG+ cells that had characteristics of FSClow at bottom left corner of the density plot. Therefore, more than 95% of cells analysed by VSG-based flow cytometry were unlikely to be cell debris. (B) Morphology of cells having characteristics of FSClow (< 50 K). P. falciparum-infected erythrocytes were subjected to flow cytometric analysis. The gated single cells were sorted according to intensity of FSC. There were ring forms and schizonts observed in the FSClow cells. Thus, inclusion of the FSClow cells is required. Microscopic images were captured using a microscope with objective lens of 100x magnification. [file 12936_2020_3156_MOESM1_ESM.pdf]

Early and mature  
schizonts

Early  
gametocytes

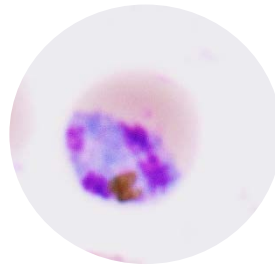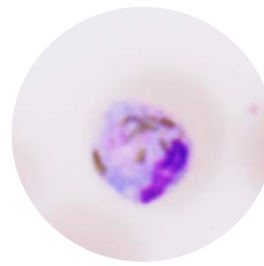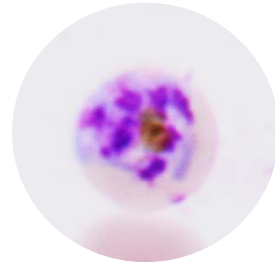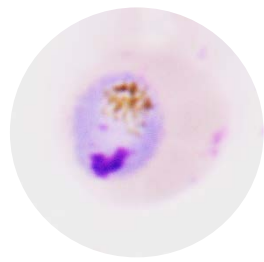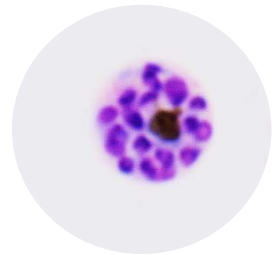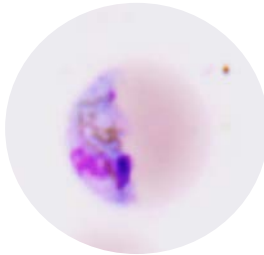

Stage IB

Stage II

10  $\mu$ m

Supplement: Supplementary file 2 — Additional file 2: Fig. S2. VSG-based flow cytometric analysis of gametocyte. Early schizonts and mature schizonts (left panel) and early stage gametocytes (right panel) were observed in VSGhigh fraction. In the VSGhigh fraction, parasitized erythrocytes have granular distribution of haemozoin, resembling stage IB. Moreover, some were elongated and D-shaped within erythrocytes, which are key characteristics of stage II gametocytes. Early schizonts having 2 and 6 divided nucleus, and mature schizonts consisting of 14 merozoites were also observed in the VSGhigh fraction, whereas ring forms and trophozoites were observed in the VSGlow and VSGintermediate fractions. Scale bar: 10 μm. [file 12936_2020_3156_MOESM2_ESM.pdf]

## VSG+ cells sorted by flow cytometer

### A. Non-synchronized

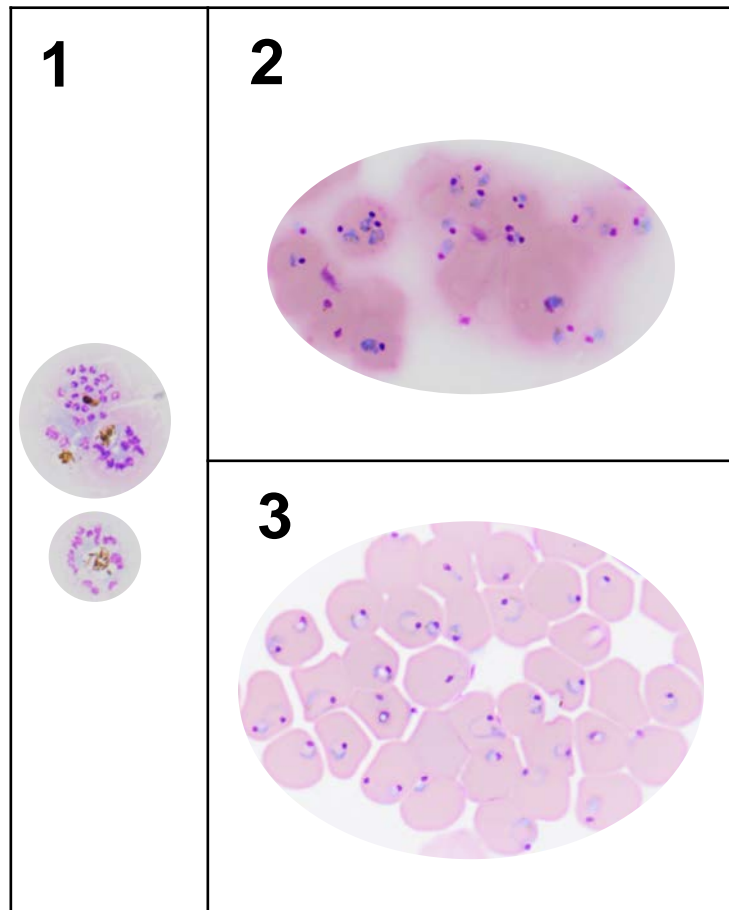

### B. Synchronized

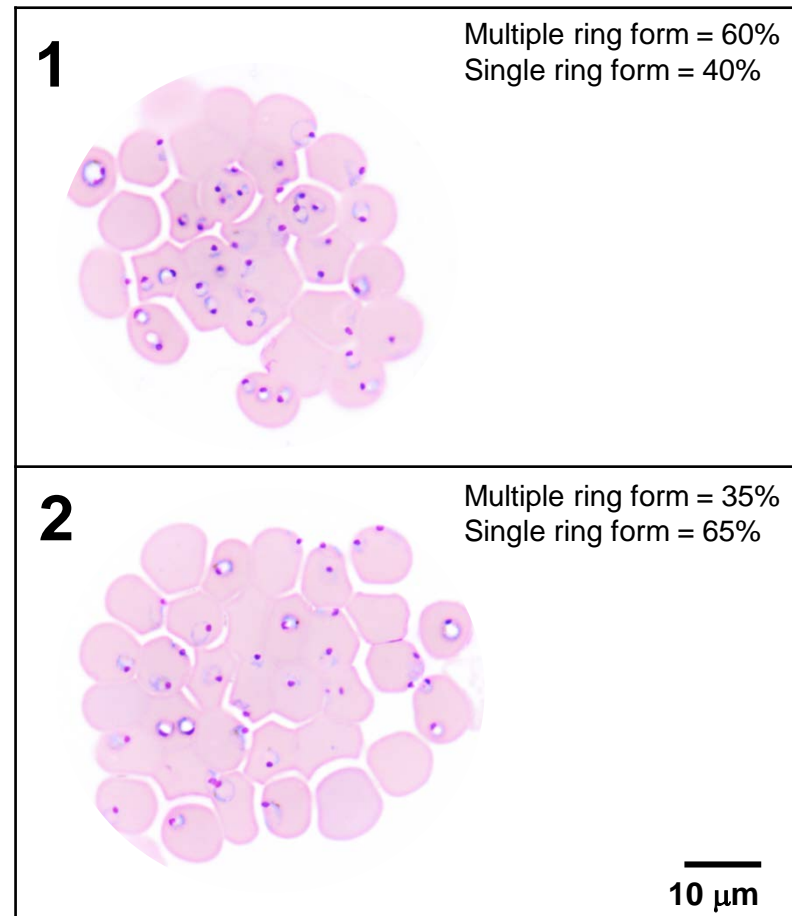

Supplement: Supplementary file 3 — Additional file 3: Fig. S3. Giemsa stain of VSG+ cells sorted from non-synchronized and synchronized culture of P. falciparum. (A) Morphology of three distinct populations observed in the non-synchronized culture (Fig. 5b, left panel): (1) cells having small size with various granularity (approximately 0–45 K of FSC-A, and 30–170 K of SSC-A); (2) cells having a relatively large size with high granularity (approximately 45–185 K of FSC-A, and 75–170 K of SSC-A); and, (3) cells having a relatively larger size with low granularity (approximately 45–185 K of FSC-A, and 20–75 K of SSC-A). Based on the intensity of VSG and microscopic images, the population number 1, 2, and 3 in the upper panel of Fig. 5b are schizonts, trophozoites, and ring forms, respectively. (B) Morphology of one minor (indicated as 1) and one major (indicated as 2) population observed in the synchronized culture (Fig. 5b, right panel): (1) cells having high granularity (more than 160 K of SSC-A); and (2) cells having with low granularity (lower than 160 K of SSC-A). Both had a similar size (50–150 K of FSC-A). Based on microscopic images, majority of the VSG+ cells with more than 160-K SSC-A are infected erythrocytes containing multiple ring forms, and they had VSG intensity of 11,578; whereas, the majority of VSG+ cells with lower than 160 K SSC-A are infected erythrocytes with single ring forms and they had VSG intensity of 10,182. [file 12936_2020_3156_MOESM3_ESM.pdf]
